# The Correlation of Sleep Disturbance and Location of Glioma Tumors: A Narrative Review

**DOI:** 10.3390/jcm12124058

**Published:** 2023-06-15

**Authors:** JuliAnne E. Allgood, Avery Roe, Bridger B. Sparks, Mercedes Castillo, Angel Cruz, Amanda E. Brooks, Benjamin D. Brooks

**Affiliations:** 1 Department of Neuroscience, University of Wyoming, Laramie, WY 82071, USA; 2 College of Osteopathic Medicine, Rocky Vista University, Greenwood Village, CO 80112, USAabrooks@rvu.edu (A.E.B.)

**Keywords:** glioma, sleep, sleep disturbance, tumor location, quality of life, cancer

## Abstract

Sleep disturbance can occur when sleep centers of the brain, regions that are responsible for coordinating and generating healthy amounts of sleep, are disrupted by glioma growth or surgical resection. Several disorders cause disruptions to the average duration, quality, or patterns of sleep, resulting in sleep disturbance. It is unknown whether specific sleep disorders can be reliably correlated with glioma growth, but there are sufficient numbers of case reports to suggest that a connection is possible. In this manuscript, these case reports and retrospective chart reviews are considered in the context of the current primary literature on sleep disturbance and glioma diagnosis to identify a new and useful connection which warrants further systematic and scientific examination in preclinical animal models. Confirmation of the relationship between disruption of the sleep centers in the brain and glioma location could have significant implications for diagnostics, treatment, monitoring of metastasis/recurrence, and end-of-life considerations.

## 1. Introduction

Sleep has well-documented physical, psychological, and behavioral benefits that can be diminished when sleep is disrupted [1]. In fact, sleep disturbance is associated with many neurological disorders including stroke, psychological dysfunction, and traumatic brain injury (TBI) [2,3,4,5]. Current guidelines recommend that adults get between 7 and 9 h of sleep per night, with children and adolescents often needing more, to maintain optimal physical and mental health [6,7]. While all of the positive benefits of sleep have yet to be proven, it is generally accepted that sleep is necessary to restore and maintain physiological homeostasis after waking periods by removing waste through the glymphatic system, initiating and maintaining macromolecule biosynthesis, maintaining prophylactic cells, and regulating metabolic functions and energy balance [8,9]. The literature is clear that a healthy amount of sleep is essential for daily function as well as the prevention and treatment of chronic health conditions, especially cancer [10]. Patients with cancer have a higher-than-normal incidence of sleep disturbance; a problem that can continue for up to 9 years after entering remission [11]. Sleep disturbance is especially detrimental to patients with gliomas, a common brain tumor that, depending on grade and location, carries a high mortality rate and poor prognosis [12,13]. Disturbance to healthy amounts of sleep in these patients impacts recovery, quality of life (QoL), and the patient’s support system.

Sleep disturbances in patients with gliomas can be a presenting symptom or a detrimental side effect of treatment. The call to address sleep disturbance, caused by tumor growth, treatment, or comorbid disorders, in patients with cancer has recently become more prevalent in the neuro-oncology literature [14]. Surveys of patients with primary brain tumors indicate that sleep disturbance is common, 61.5% of patients, and that their symptoms were not well-addressed or treated by their doctors [15]. While this indicates the magnitude of the problem for patients, it also shows a potentially facile way to track glioma progression that is currently overlooked. 

When looking at the anatomic localization of gliomas and neurologic impairment, one study conducted by Sadighi et al. found that tumor location changed the severity and type of neurologic impairments caused by tumor growth [16]. This study showed that, in addition to other symptoms, specific sleep disturbances might vary by patient, but the variation was contingent on the tumor’s location [16]. Glioma location is not just a consideration in anatomical correlation studies, it is also a top consideration when developing a treatment plan. The potential for surgical resection is affected by accessibility to the tumor and the regional function of the brain in relation to the tumor location. The highly organized structure of the brain can be beneficial for these surgical considerations, but it can also allow for more pointed diagnostic exploration based on presenting symptomatology [17]. The brain regions attributed to the normal functioning of sleep, or sleep centers, can be affected by tumoral growth directly or by neuroinflammation and edema from growing gliomas [18]. Sleep centers disrupted by glioma growth could provide clues as to the initial location of a glioma, as a consideration for surgical resection, or as a potential tool to track recurrence. The correlation between sleep disturbance and glioma location has been noted in a number of case reports subsequently discussed in this text, but its merits in treatment and as a warning symptom of metastasis or recurrence have yet to be explored. Reliable correlations of sleep disorders with glioma growth in sleep centers have yet to be verified or studied in preclinical animal models or large-scale clinical audits. 

This narrative review will specifically focus on gliomas located in or near brain areas documented to impact sleep function. Case reports were compiled from multiple sources using a wide range of search terms in an attempt to identify as many cases as possible where sleep disturbance can be correlated with glioma growth. While there are numerous sleep disturbances, the scope of this narrative review is limited to insomnia, hypersomnia, sleep apnea, narcolepsy, and somnolence as they are the sleep disorders that are most heavily correlated with glioma. Consideration will be especially given to high-grade gliomas as they are more aggressive than low-grade gliomas. The higher mortality rate associated with high-grade gliomas highlights the potential sleep consequences of maximal surgical resection and treatment as well as shows the importance of maintaining healthy levels of sleep near the end of life to maintain QoL. 

## 2. Brain Areas Implicated in Sleep Disturbance

**Figure 1:**
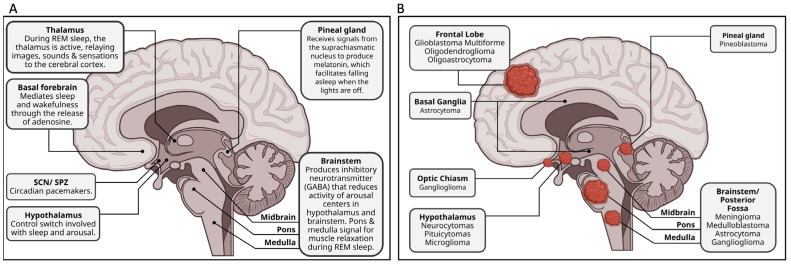
Schematic depiction of brain areas implicated in sleep and common glioma locations. Midsagittal depiction of the brain identifying common brain areas implicated in sleep (**A**) and the locations of gliomas documented in this review (**B**).

Normal sleep arises from a coordination of sleep centers in the brain, such as the hypothalamus, pineal gland, and the brainstem (Figure 1A) [1]. Sleep induction largely follows a well-described path. Initially, specific retinal ganglion cells that contain melanopsin detect a decrease in ambient light [19]. This photosensitive protein within the retinal ganglion cells will project to the retinohypothalamic tract, stimulating the suprachiasmatic nucleus (SCN) [20]. The SCN and subparaventricular zone (SPZ), which share strong neuronal connections and are responsible for circadian control, keep a 24 h cycle by way of a transcriptional–translational feedback loop [21]. Inside the SCN neurons there are two genes, BMAL1 and Clock, which, when transcribed, create Per and Cry proteins. Per and Cry proteins leave the nucleus and build up to a critical concentration where they then re-enter the nucleus and inhibit the translation of BMAL and Clock [21,22]. This process takes 24 h and allows the SCN to keep the body’s cells under tight circadian control. The SCN also releases norepinephrine which stimulates the release of melatonin from the pineal gland to induce sleep [23].

In coordination with the hypothalamus and pineal gland, other brain regions also play significant roles in the initiation, perpetuation, and conclusion of sleeping periods. For example, the preoptic area of the hypothalamus and thalamus coordinate sleep and waking states, while further sleep regulation and induction are influenced by melatonin secreted from the pineal gland [24]. This provides time-of-day information to all cells and serves as a biomarker of the central biological clock [10]. The hypothalamus and the adjacent neuron groups of the basal forebrain regulate sleep duration by producing GABA. The main function of these GABAergic neurons is to inhibit the firing of cells involved in wakefulness [25]. Histamine, norepinephrine, serotonin, hypocretin, and glutamate neurons are inhibited by GABA to promote sleep. GABAergic neurons are more active during nonrapid eye movement (NREM) sleep as opposed to other neurons that are active during more wakeful rapid eye movement (REM) sleep. Neurons in the hypothalamus and basal forebrain increase the rate of GABA discharge during sleep onset and will continue to release GABA at a high level while sleep continues [25].

The brainstem, particularly the PONS, also has a major role in sleep [26]. While there is still some uncertainty surrounding the exact purpose of the PONS, it is known that there are many neuron types (cholinergic, noradrenergic, glutamatergic, and GABAergic) in the PONS that are key to generating REM sleep [27]. The brainstem also controls sleep through projections to the lateral hypothalamus, which contain many hypocretin-containing neurons [28]. Hypocretin is a neuropeptide that functions to regulate arousal and wakefulness. These hypocretin-containing neurons will then project to the thalamus, which has widespread projections throughout the brain [29]. 

While the importance of sleep to normal homeostasis and cancer pathology is continuing to be unraveled, exploring sleep centers and correlations to sleep disturbance is a large field of research. Sleep disturbance can present in many forms when caused by tumors or strokes located in brain areas necessary for coordinated sleep [30]. For gliomas specifically, sleep disturbance can be an initial symptom used in diagnosis, a developed symptom due to metastasis or recurrence, or as a side effect of treatment (surgical, radio, or chemopharmaceutical) and there are many ways in which tumors can interfere with sleep [14]. 

Direct destruction by tumor growth is one way tumors can impact brain function related to sleep. Destruction of sleep centers can lead to lifelong sleep disturbance when the tumor mass damages neuronal tissue or when the tumor mass is removed [31]. Alternatively, based on similar damage seen in patients with TBI, sleep disturbance can potentially result from neural damage not directly caused by tumor cell infiltration such as hypoxia, which can alter pH, and edema, which can cause compression of neural tissue [32,33,34]. Stroke studies in which irreversible neuronal death caused long-term sleep disturbance and cytotoxicity, neuronal death caused by gliomas can also lead to long-term, sometimes irreversible, sleep disturbances [35]. Discovering the underlying cause of sleep disturbance will aid in determining the appropriate treatment for each patient. For patients with glioma, removal of the tumor and preservation of speech, motor, and life-sustaining brain areas takes precedence to the maintenance of sleep centers, leaving patients with limited, often ineffective, treatment options for sleep disorders that include medication and sleep hygiene education [36]. 

## 3. Characterization and Treatment of Gliomas

The Centers for Disease Control and Prevention and the National Cancer Institute created the Central Brain Tumor Registry of the United States (CBTRUS), a professional research organization dedicated to providing quality statistical and descriptive epidemiological data on primary brain and other central nervous system tumors newly diagnosed in the United States population. They published their latest report of incidence rates according to behavior (i.e., malignant and nonmalignant), histopathology, age, sex, race, select brain molecular markers (BMM), Hispanic heritage, and geographical location in October of 2022. According to CBTRUS, nonmalignant tumors are over two times more likely to occur (72% vs. 28%), but the five-year survival rate for malignant brain tumors is only 35.7% [37]. This value decreases to 6.8% for glioblastomas, a term for all grade IV gliomas. 

Gliomas are graded and characterized according to the WHO classification system. The WHO classification system for brain and CNS tumors assigns a grade to the glioma from I to IV, with I being the least aggressive (often considered nonmalignant) and IV being the most aggressive [38]. High-grade gliomas fall into grades III or IV. Importantly, WHO classification grades are based on the appearance of the tumor cells, a characterization impossible to achieve with radiographic confirmation alone. Specifically, classification is based on five histopathology traits: cellular density, nuclear atypia, mitosis, endothelial proliferation, and necrosis (Supplemental Table S1) [39]. In 2016, the WHO classification scale for tumors of the central nervous system included brain molecular markers, or biomarkers, for the first time. These markers have improved in their fidelity from 2018 to 2019 and continue to help clarify glioma grading, which can have a significant impact on treatment and prognosis. 

**Figure 2:**
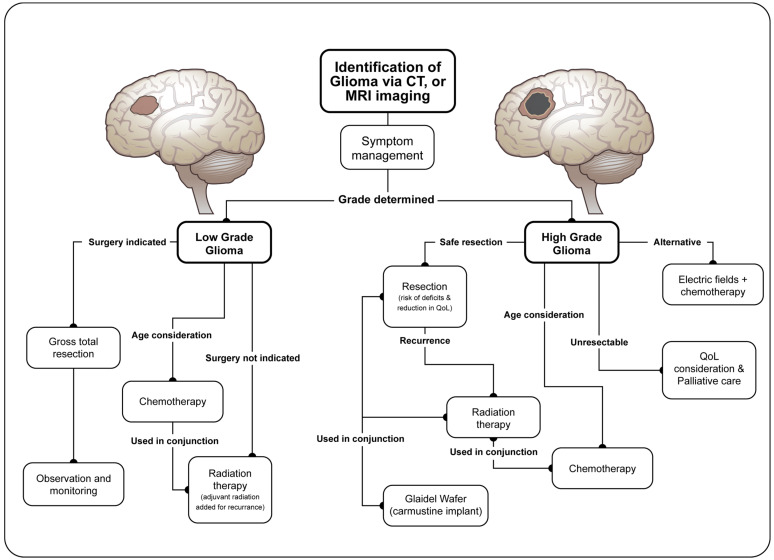
Current treatment course for glioma tumors in the United States. Flow chart showing the path of initial identification of glioma through the treatment options based on glioma grade. Treatment information for flow chart taken from UpToDate [41].

Although biomarkers are helping, there is no singular definition of glioma: they are characterized only as arising from glial precursor cells. Such tumors include glioblastoma, astrocytoma, oligodendroglioma, ependymoma, oligoastrocytoma (mixed glioma), and a few rare histopathologies with glioblastomas that cumulatively account for slightly over 50% of malignant tumors in the brain [40]. Figure 1B indicates the glioma tumor types associated with sleep disturbance that were documented in this review. In the United States, identifying a glioma tumor traditionally begins through imaging, usually via CT or MRI (Figure 2) [41]. Despite significant advances, imaging alone cannot define if a mass is a cancerous tumor, and a further diagnostic work-up requires surgical biopsy. Classically, surgery is performed for increased accessibility to specific areas of the brain, allowing for pathological evaluation of the mass and simultaneous resection. In more critical areas of the brain, such as near the brainstem, a stereotactic needle biopsy is taken to identify tumor morphology before surgical resection is performed [42]. Histopathology of biopsied cells is necessary for grading the tumor and determining the course of treatment. 

Once a glioma has been identified and graded, symptomatic management is the primary goal as curative options, aside from a successful complete surgical resection, are generally unavailable (Figure 2). Presenting symptoms of gliomas include headaches, seizures, nausea, vomiting, change of vision, ambulatory difficulty, weakness, tingling sensations, and new or worsening sleep disorders [43,44]. Patients most commonly present with headaches followed by seizures which are hypothesized to be caused by increased pressure among the microvasculature, resulting in edema [45]. Most symptoms are specific to the areas of the brain in which the tumor is proliferating [44]. For example, patients with tumors proliferating in the temporal lobe can be identified by changes in receptive speech issues and patients presenting with focal or generalized seizures tend to have tumors in the cerebral cortex [46]. 

First-line treatment for gliomas is surgical resection, where as much tumor material as possible is removed (Figure 2) [41]. Tumor location is one of the most important factors to consider before the resection of a glioma. Consideration of the extent of resection and the consequences to the function of surrounding or removed brain tissue is of utmost importance to patients and surgeons. While attempts are made to reduce the risk of neurological deficits, such as functional sleep and reduction in QoL, maximal resection of tumor tissue is the ultimate goal to improve prognosis. As such, patients and surgeons often accept the risks of neurological deficits and a reduction in QoL before surgery is attempted. Nevertheless, many patients also rate sleep disturbance as a high area of concern during treatment, despite it often being a low priority for providers [43]. 

Radiation therapy is another treatment option used when surgery cannot be performed, or when the tumor is high-grade, and can help to delay recurrence and prolong survival (Figure 2) [41]. Effective radiation treatment is aimed at the original tumor location with a small surrounding margin because recurrence usually happens within 2 cm of the original tumor [47]. Chemotherapy is another choice to treat gliomas and can be used alone or in conjunction with radiotherapy (Figure 2) [41]. Chemotherapeutics are aimed at slowing or stopping neoplastic proliferation. Chemotherapeutic treatment must take into account a patients’ age since adults, whose neural tissues are not growing rapidly, are more protected from the chemotherapeutic side effects than pediatric patients where damage of actively growing tissue can lead to more severe side effects (Figure 2) [48]. It is worth noting an emerging treatment in the form of electric fields, where a battery-powered device provides low-strength electric fields around a tumor. This device needs to be worn for a minimum of 18 h per day and has been shown to increase survival when used alongside chemotherapy in patients with grade IV gliomas [49].

In patients with high-grade gliomas, and those in which a recurrent tumor is found, QoL is an important consideration since the median survival is significantly reduced (15 months for a grade IV glioma) [50]. QoL management becomes gradually more difficult with disease progression because the development of psychiatric conditions such as depression and anxiety as well as the development of new cognitive impairment can increase the difficulty of symptom triage and management [51]. Song et al. demonstrated that this development of cognitive issues can also be tied to systemic inflammation via IFN-y and IL-2 and not solely tumor location [51]. Development of these comorbid conditions can cause patients to present with new or worsening sleep disorders requiring new treatment and QoL management. 

## 4. Correlation of Tumor Location and Sleep Disturbance

Among the top concerns for neurosurgeons confronted with a glioma is tumor location [52]. There are several sleep centers in the brain that rely on coordinated communication to generate, perpetuate, and terminate sleep. Understanding of the neurophysiological pathways used to generate sleep and the associated brain regions key to these processes is beneficial when treating patients with glioma. With this understanding, better diagnostic and prognostic decisions can be made that consider the patient’s QoL, overall survival, and proper course of treatment. Presenting sleep disturbance symptoms can potentially be one of the first clues pointing to a glioma location. Sleep apnea, hypersomnia, narcolepsy/cataplexy, parasomnia, and insomnia have been linked with tumor location in several case reports and retrospective chart analyses outlined in Table 1. 

### 4.1. Sleep Apnea

Sleep apnea as a presenting symptom is implicated in a large number of case reports where gliomas have been identified [53,54,55,56,57,58,59,60,61,62,63,64]. There are two main types of sleep apnea, central and obstructive (OSA), with polysomnography testing being required for diagnosis [58]. A number of glioma types have a reported association with sleep apnea, with tumors mainly being located in the PONS and medulla or occasionally the frontal lobe. Those located in the medulla can in some cases cause a very specific set of symptoms known as Ondine’s curse, a failure of automatic respiration during the night in which assisted ventilation or death are the prevailing outcomes [78]. This is commonly reported in cases of brainstem tumors in children but can be found in adult cases as well. For example, in a case report by Nakajima et al., a 49 year old woman was found in respiratory arrest that required resuscitation [60]. MRI revealed a glioma in the lower PONS and medulla with subsequent testing revealing Ondine’s curse, in which her autonomous respiration ceased during sleep. Treatment required two months of respiratory support and radiation therapy for the glioma. 

While tumoral disruption of the medulla and PONS are the overwhelming cause of tumor-associated sleep apnea, tumors in the frontal lobe have also been correlated with sleep apnea, specifically OSA. In a drastically different mechanism to the brainstem, tumor growth in the frontal lobe disrupts control of the phrenic and intercostal musculature nerves, reducing the motor function necessary to take breaths [53]. Tumors in the frontal lobe and brainstem also result in other serious motor deficits that can obscure the discovery of sleep apnea as a presenting symptom. In contrast to other sleep disorders that can potentially mimic or be caused by other consequences of a poor night’s sleep, sleep apnea is a relatively unique and easily definable disorder. As a result of this, identification of sleep apnea as a presenting or recurring symptom of glioma is more readily accomplished by those without expertise in sleep medicine.

### 4.2. Hypersomnia

Diurnal drowsiness or hypersomnia is another condition that can be correlated with gliomas found in the posterior fossa, hypothalamus, and thalamus/midbrain [57,63,64,65,66,67]. Hypersomnia is characterized by excessive daytime sleepiness that can impact academic or work performance. Hypersomnias are tracked, diagnosed, and classified by their reported sleep quality, diurnal sleep, and the overall restfulness a patient feels after sleep [79]. Somnolence is also another term that falls under the umbrella of hypersomnia and describes a strong desire to fall asleep or constant state of drowsiness that has been well-characterized as a side effect of radiation therapy [80]. A specific case report by Anderson and Salmon details a 23-year-old man who had hypersomnia as a presenting symptom [66]. The hypersomnia seen in this patient progressed to narcolepsy, followed by attacks of sleep paralysis. Exploration of the brain found a glioma in the right-side hypothalamus. No treatment was given for the glioma, but medications to aid with nocturnal sleep were given with no success. Diurnal drowsiness and hypersomnias can be difficult to characterize because they need to be separated from other sleep or mood disorders with extensive testing [81]. Once it is clear that excessive diurnal sleepiness is negatively impacting everyday living, or that a sudden/unusual onset of diurnal sleepiness has occurred, the presentation of this symptom can indicate an underlying condition such as glioma. Treatment of hypersomnia in glioma patients includes medication or scheduled daily naps to improve daily functioning. 

### 4.3. Narcolepsy

Narcolepsy is another presenting symptom in patients with glioma. This is especially well researched in pediatric cases and is often associated with concurrent cataplexy. Narcolepsy, which can either be type 1 (with cataplexy and low levels of hypocretin) or type 2 (without cataplexy and normal hypocretin), is excessive daytime sleepiness affecting daytime functioning [82]. Narcolepsy with cataplexy results in sudden loss of muscle tone due to strong emotions [82]. Case reports show that the onset of narcolepsy can be correlated with tumoral infiltration in or near the hypothalamus, optic chiasm, sellar and suprasellar region, and hippocampus [66,67,68,69]. Among the top consequences for this type of sleep disturbance are poor QoL and reduced academic success for children where narcolepsy impacts their ability to function normally. Rosen et al. described 14 cases of children aged 5 months to 15 years, both males and females, detailing narcolepsy symptoms in many different types of gliomas located in the optic chiasm, brainstem, pituitary gland, pineal gland, frontal lobe, and posterior fossa. Treatment of gliomas included gross total resection, radiation treatment, chemotherapy, and shunt placement depending on the individual patient. Treatment for narcolepsy symptoms in these patients was heavily focused on stimulant medications. This retrospective chart review, along with other case reports of glioma, germinoma, stroke, and TBI discussed in later sections, provide excellent evidence to suggest that presenting narcolepsy symptoms can be varied but are often localized to only a few deep brain regions. 

Treatment of narcolepsy in glioma patients is based on stimulant medication used to reduce excessive daytime sleepiness. One case of glioma with concurrent narcolepsy found that stimulant medication is effective at treating narcolepsy symptoms, but required a larger than normal dose with symptoms returning when the medication is stopped [70]. A trial of a popular stimulant medication, modafinil, in primary tumor patients found that symptoms were not improved compared to placebo patients [83]. These results indicate that the effects from stimulant drugs could potentially be dose-dependent or be impacted by tumor location and severity; an interesting consideration requiring more research for clinicians treating glioma patients.

### 4.4. Parasomnias

Parasomnias are a set of sleep disorders that include night terrors, sleepwalking, or sleep paralysis and sleep-related eating disorders [84]. Various parasomnias have been reported in patients with gliomas of the brainstem, thalamus, or parietal lobe [66,71,72,73]. For example, a case reported by Gennaro et al. described a suspected glioma in the right thalamus of a 48-year-old woman [72]. She presented with night terrors in which she would sit up in bed, scream, and was agitated and unresponsive. While the patient refused biopsy and treatment for the glioma, medication for the night terrors successfully reduced her episodes. Treatment of parasomnias is unique to the type of disorder but generally includes medication or behavioral therapy. Medication can be ineffective for those experiencing parasomnias, so most often therapies are recommended in addition to medication. Psychotherapy, relaxation therapy, and autogenic training or hypnosis are recommended to treat parasomnias. These behavioral therapies are effective for the treatment of parasomnias with comorbid disorders, such as cancer, without impacting cancer treatments [85]. Behavioral therapy treatment for sleep disorder in glioma patients is more complicated because the location of the tumor can create cognitive, mood, or other psychiatric disorders that can complicate the therapy necessary to treat sleep disorders [86]. Parasomnias are unique to each patient in presentation and treatment, but if properly tracked can be a good indicator of dysfunction when they occur with sudden onset or with an increase in the number, duration, or severity of episodes. 

### 4.5. Insomnia

Insomnia, common in the general population, has also been noted as a presenting symptom in patients with gliomas [74,87,88]. However, insomnia is difficult to correlate with tumor location alone since it is commonly associated with neuropsychiatric disorders and the general anxieties associated with cancer diagnosis [89]. It is also important to note that insomnia is often induced by the treatment of glioma and does not have to be a presenting symptom. One such case report of a 29-year-old female described by Reim et al. found that radiation therapy of a glioma in the basal ganglia caused severe insomnia that was not present before treatment [74]. The sleep medication Nitrapasepam did not improve the patient’s insomnia, but melatonin supplements alone were able to reverse her symptoms. Insomnia is often a comorbidity seen in patients with cancer due to the anxiety and fear that accompanies diagnosis and does not necessarily indicate a brain tumor [90]. The key for understanding insomnia as a symptom of glioma is the onset, severity, and the number of episodes in correlation with other glioma symptoms. Treatment of insomnia in glioma patients includes medications, which can be ineffective, or cognitive behavioral therapy, which has shown promise in clinical trials for improving sleep in glioma patients [91]. 

### 4.6. Other Brain Disorders

Sleep disturbances are also correlated with other primary brain tumors, such as hemangioblastomas. Case reports have found sleep apnea in cases of hemangioblastomas located in the medulla and 4th ventricle as well as sleepwalking in dysembryoplastic neuroepithelial tumors and narcolepsy in a patient with a germinoma (Table 1) [75,76,77]. Additionally, evaluation of pediatric patients with uncharacterized brain tumors in the hypothalamic/pituitary brain regions that underwent surgical resection reported severe hypersomnolence following surgery [92]. This indicates that iatrogenic disruption of the sleep centers during glioma removal is also an important and often under-considered consequence of treatment that should potentially be further discussed by surgeons and patients before intervention. 

While there are relatively few care reports of patients with gliomas and sleep disturbance, TBI and stroke patients are known to have sleep disorders as a result of their injuries. For example, TBI patients where sleep disturbance has developed as a consequence of the injury are well-documented and more completely studied than tumor or stroke patients [30,32,93,94,95,96,97]. One study looking into sleep disturbances as a potential marker of brain injury in patients with mild TBI found that there were abnormalities in the polysomnography results [98]. The patients in this study suffered from OSA and/or restless leg syndrome, but no data indicating the location of injury were obtained. Patients that have had a stroke represent an interesting population to study sleep disturbance in because certain sleep disorders can be a risk factor for a stroke, or these disorders can result from the damage caused by the stroke itself [99]. Hypersomnolence secondary to a stroke has been studied clinically and found to be most closely associated in individuals with para median thalamic infarctions [100]. Cases of tumor growth, stroke, and TBI provide additional support for the idea that there is a potentially facile correlation of tumor growth and sleep disturbance that merits further exploration.

## 5. Future Considerations

As holistic treatment approaches grow, sleep disturbances associated with glioma could offer important diagnostic and remission monitoring opportunities. As such, this is a promising research area. With an increased focus on the patient’s QoL, examination of the causes and consequences of cancer-associated sleep disturbances is increasing. A mechanistic understanding of the physical structure of the brain’s sleep centers, as well as a molecular understanding of the neuronal circuits associated with those regions, may help guide both surgical and other treatment approaches. Compiling a complete understanding of the consequences of surgery is incredibly valuable to both the patient and the surgeon as they decide if the benefits of glioma resection outweigh the risks. Future research on this topic should include consideration of the impact of both tumor location and tumor treatment on the sleep centers of the brain. Important questions include how the underlying glioma growth impacts the type and severity of sleep disturbance, the consequences of tumoral growth in or near key sleep centers of the brain, and the benefits and costs of complete surgical resection when tumors are located close to sleep centers. 

## 6. Conclusions

Sleep is considered important for mental and physical health. There are many benefits to maintaining healthy amounts of sleep including hormonal balance and removal of toxins and metabolic waste by the glymphatic system [101]. Additionally, sleep has been shown to increase energy levels and improve cognitive function and memory [7]. These benefits can be marred by sleep disturbances, which evoke physical, mental, and emotional anguish. In several cancer types, sleep disturbance is listed as one of the most distressing and debilitating effects of the cancer itself and treatment [102]. 

The mechanisms that can cause sleep disturbances in patients with cancer are varied, including direct disruption to a sleep center, hypoxia induced by tumor growth, or edema created by the growing tumor mass. The direct implications of this mechanism can alter the treatment outcome and be used to understand the correlation between glioma location and sleep disturbance. As shown in Table 1, a number of case reports identify this correlation and highlight the importance of future animal and clinical studies to verify validity. This type of data-driven exploration would allow for sleep disorders to be considered more heavily at initial diagnosis and used as warning signs of recurrent or metastatic glioma. Furthermore, a strong correlation between tumor location and specific sleep disorders could have implications for surgical resection, medications, and QoL recommendations for patients with glioma. Ultimately, sleep is a relatively easy physiological process to monitor and as such could be one way to continuously monitor high-risk patients for signs of recurrent or new growth. 

## Figures and Tables

**Table 1:** Cases with brain tumor diagnosis and sleep disturbance.

Author	Year	Patient Age	Patient Gender	Tumor Location	Tumor Type	Sleep Disturbance
Discolo et al. [53]	2005	71	Male	Right frontal lobe	Glioblastoma Multiforme	Sleep apnea
Osanai et al. [54]	1994	44	Male	Left cerebellar peduncle, medulla	Ganglioglioma	Sleep apnea
Ioos et al. [55]	2016	4	Female	Posterior fossa	Meningioma	Sleep apnea
Greenough et al. [56]	1999	34	Male	Bilateral medulla	Medulloblastoma	Sleep apnea
Manning and Leiter [57]	2000	18	Female	Medulla	Ganglioglioma	Somnolence, sleep apnea
Ito et al. [58]	1996	12, 6	Male	Medulla, pons	Glioma	Sleep apnea
Kelly et al. [59]	1980	4 weeks	Male	Left middle fossa	Astrocytoma	Sleep apnea
Nakajima et al. [60]	2000	49	Female	Medulla, pons	Astrocytoma	Sleep apnea (Ondine’s curse)
Marin-Sanabria [61]	2005	52	Female	Medulla	Glioma	Sleep apnea (Ondine’s curse)
Huang et al. [62]	2021	4	Male	Medulla, pons	Glioma	Sleep apnea
Hui et al. [63]	2000	3	Male	Posterior fossa	Pilocytic astrocytoma	Hypersomnia, sleep apnea
Valente et al. [64]	1993	24	Male	Pons	Fibrillary astrocytoma	Sudden awakenings, hypersomnia, sleep apnea
Yen et al. [65]	2022	48	Male	Right side thalamus and midbrain	Glioma	Hypersomnia
Anderson et al. [66]	1977	23	Male	Hypothalamus	Glioma	Narcolepsy, hypersomnia, sleep paralysis
Liao et al. [67]	2020	44	Male	Hippocampal formation	Glioma	Somnolence, Narcolepsy type 2
Laus et al. [68]	2022	3	Male	Optic chiasm	Ganglioglioma	Somnolence, Narcolepsy
Rosen et al. [69]	2003	5–15	Males and Females	Hypothalamus, optic chiasm, brainstem, pineal gland, pituitary gland	Pineoblastoma, craniopharyngioma, medulloblastoma, astrocytoma	Narcolepsy
Butts et al. [70]	2014	53	Male	Corpus callosum	Glioma	Somnolence, Narcolepsy
Mendez [71]	1992	15	Male	Fourth ventricle and brainstem	Astrocytoma	Night terrors
Di Gennaro et al. [72]	2004	48	Female	Right thalamus	Patient refused biopsy	Night terrors
Duffau et al. [73]	2006	38	Female	Right paralimbic region	Oligodendroglioma	Epileptic somnambulism
Reim et al. [74]	2016	29	Female	Right basal ganglia	Astrocytoma	Insomnia
Fukushima et al. [75]	1998	16, 33	Female, Male	Medulla	Hemangioblastoma	Sleep apnea
Prashad. [76]	2013	15	Male	Parietal lobe	Dysembryoplastic neuroepithelial tumor	Sleepwalking
Weil et al. [77]	2017	11	Female	Sellar and suprasellar region	Germinoma	Narcolepsy

## Data Availability

Not applicable.

## Supplementary Materials

The following supporting information can be downloaded at: https://www.mdpi.com/article/10.3390/jcm12124058/s1, Table S1—WHO glioma grading based on histopathology traits.
