# Supplementary material for: The Correlation of Sleep Disturbance and Location of Glioma Tumors: A Narrative Review
Source: J Clin Med. 2023 Jun 15;12(12):4058. doi: 10.3390/jcm12124058 (PMC10298947; doi:10.3390/jcm12124058)
Supplement: Supplementary file 1 [file jcm-12-04058-s001.zip › jcm-2403453-supplementary.pdf]

Table S1- WHO glioma grading based on histopathology traits.

Grade	Cellular Density	Nuclear atypia	Mitosis	Endothelial Proliferation	Necrosis
**II**	+	Occasional	Absent	Absent	Absent
**III**	++	Distinct	Marked	Absent	Absent
**IV**	+++	Marked	High	Present	Present
